# Calcium Permeable Channels in Cancer Hallmarks

**DOI:** 10.3389/fphar.2020.00968

**Published:** 2020-07-07

**Authors:** Sendoa Tajada, Carlos Villalobos

**Affiliations:** Instituto de Biología y Genética Molecular (IBGM), Universidad de Valladolid and Consejo Superior de Investigaciones Científicas (CSIC), Valladolid, Spain

**Keywords:** Ca^2+^ channels, cancer hallmarks, store-operated Ca^2+^ entry, TRP channels, calcium channel modulators in cancer

## Abstract

Cancer, the second cause of death worldwide, is characterized by several common criteria, known as the “cancer hallmarks” such as unrestrained cell proliferation, cell death resistance, angiogenesis, invasion and metastasis. Calcium permeable channels are proteins present in external and internal biological membranes, diffusing Ca^2+^ ions down their electrochemical gradient. Numerous physiological functions are mediated by calcium channels, ranging from intracellular calcium homeostasis to sensory transduction. Consequently, calcium channels play important roles in human physiology and it is not a surprise the increasing number of evidences connecting calcium channels disorders with tumor cells growth, survival and migration. Multiple studies suggest that calcium signals are augmented in various cancer cell types, contributing to cancer hallmarks. This review focuses in the role of calcium permeable channels signaling in cancer with special attention to the mechanisms behind the remodeling of the calcium signals. Transient Receptor Potential (TRP) channels and Store Operated Channels (SOC) are the main extracellular Ca^2+^ source in the plasma membrane of non-excitable cells, while inositol trisphosphate receptors (IP_3_R) are the main channels releasing Ca^2+^ from the endoplasmic reticulum (ER). Alterations in the function and/or expression of these calcium channels, as wells as, the calcium buffering by mitochondria affect intracellular calcium homeostasis and signaling, contributing to the transformation of normal cells into their tumor counterparts. Several compounds reported to counteract several cancer hallmarks also modulate the activity and/or the expression of these channels including non-steroidal anti-inflammatory drugs (NSAIDs) like sulindac and aspirin, and inhibitors of polyamine biosynthesis, like difluoromethylornithine (DFMO). The possible role of the calcium permeable channels targeted by these compounds in cancer and their action mechanism will be discussed also in the review.

## Introduction

Cancer is the second leading cause of death globally, causing 9.6 million deaths in 2018 with an increasing estimation of 70% over the next twenty years. There are more than 100 cancer types, affecting any part of the body in both sexes. However, the most common cancer types in women are: breast (25.4%), colorectal (9.6%), lung (8.8%), cervix (6.9%) and thyroid (6.3%), meanwhile lung (15.5%), prostate (14.5%), colorectal (11.4%), liver (6.8%) and bladder (4.8%) are the most common among men (https://www.wcrf.org); (https://www.who.int). Every type of cancer is characterized by its unique clinical features, molecular markers and a disease-specific profile of genes. Nevertheless, all cancer types meet several common criteria, known as the “cancer hallmarks” described in 2000 by Hanahan and Weinberg (Hanahan and Weinberg, 2000). Although, new emerging cancer hallmarks were added in the last decade, the most accepted hallmarks are: (1) sustained proliferation, (2) cell death resistance, (3) tissue invasion and metastasis and (4) persistent angiogenesis.

Since the first hypothesis by the mid-1800s, that “canalis” are present in biological membranes (Brücke, 1847), numerous physiological functions have emerged mediated by ion channels, ranging from control cellular ionic homeostasis to sensory transduction. Consequently, ion channels perform key roles in human physiology and it is not a surprise the increasing number of evidences connecting channels disorders with a broader human diseases. Nowadays, abundant evidence demonstrate that calcium, calcium permeable channels and calcium signals, play important functions in cancer cells proliferation, apoptosis resistance, invasion and drug resistance, common cancer hallmarks. The first direct evidences connecting calcium channels and cancer were published in the 1980s, when independent studies confirmed the inhibition of cancer growth using particular calcium channels blockers (Lee et al., 1988; Batra and Alenfall, 1991; Taylor and Simpson, 1992). The ion channels research not only confirm the calcium channels correlation with cancer. Other ion channels with important roles in membrane potential or cell volume regulation, including Na^+^, K^+^ and Cl^−^ channels have been involved in the development of one or more cancer hallmarks (Pardo, 2004; Wang, 2004; Kunzelmann, 2005; Wulff et al., 2009; Schwab et al., 2012; Prevarskaya et al., 2018). At the present day, the relationship between altered expression and/or function of particular ion channels and cancer is well stablished, so well that the term “oncochannels” has been recently introduced (Huber, 2013).

This review focus in the role of calcium permeable channels signaling in cancer: from molecular mechanisms to therapeutics. Even though cancer therapies and drugs improved significantly in the last years, new and more efficient treatments are still needed to fight against such indiscriminate disease. Calcium channels and transporters are widely expressed in the plasma membrane of carcinoma cells where they may represent a good therapeutic target. For this reason, the study of ion channels role in both initial and advanced stages of the disease, should be useful to explore the possible clinical value of this membrane proteins as novel targets for therapy.

In this review we will consider the role of calcium permeable channels signaling in cancer with special attention to the mechanisms behind the remodeling of the calcium signals. The calcium channel modulators and their therapeutic use in treating cancer disease will be also discussed in the review.

## Calcium Permeable Channels In Plasma Membrane, Endoplasmic Reticulum and Mitochondria

**Figure 1:**
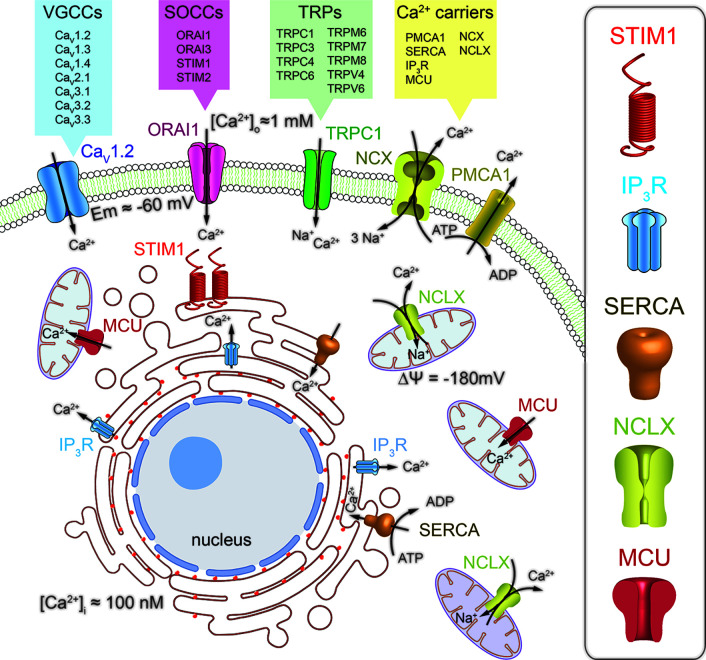
Schematic representation of the main calcium signaling pathways through Ca^2+^ channels and transporters involved in the regulation of calcium homeostasis in mammalian cells. The calcium influx pathway from the extracellular space is mediated by the combined action of the voltage gated calcium channels (VGCCs), the store-operated calcium channels (SOCCs) and associated proteins (STIM) and the transient receptor potential (TRPs) Ca^2+^ permeable channels. The calcium release pathway from the endoplasmic reticulum (ER) and mitochondria is mediated by the inositol triphosphate receptors (IP_3_R) and the mitochondrial Na^+^/Ca^2+^ exchanger (NCLX), respectively. Furthermore, the sarcoplasmic reticulum Ca^2+^-ATPase (SERCA) and the mitochondrial Ca^2+^ uniporter (MCU) are the responsible for the free cytoplasmic Ca^2+^ sequestration into organelles. Finally, the Ca^2+^ extrusion pathway from the cytoplasm is regulated by the plasma membrane Ca^2+^ATPase (PMCA1) and the Na^+^/Ca^2+^ exchanger (NCX).

Calcium permeable channels are membrane proteins located in the external and internal cell membranes, including plasma membrane (PM), and endoplasmic reticulum (ER) or mitochondria, respectively. These channels diffuse passively Ca^2+^ ions down its electromechanical gradient from extracellular space and from intracellular calcium stores to the cytoplasm. At the PM there is a wide diversity of calcium channel types, characterized by their activation mechanism: (1) Voltage-gated calcium channels (VGCCs), (2) receptor-operated calcium channels (ROCCs), (3) store-operated calcium channels (SOCCs), (4) transient receptor potential channels (TRPs), (5) acid-sensing ion channels (ASICs) and (6) stretch-activated ion channels (SAICs). VGCCs are the major participants in the Ca^2+^ entry mechanism in excitable cells, including neurons, different types of muscle cells and some endocrine cells. In contrast, the main pathway for the Ca^2+^ influx in non-excitable cells such as epithelial cells from most carcinomas is performed by SOCCs (**Figure 1**). These channels generate the store-operated calcium entry (SOCE), originally called capacitative calcium entry. SOCCs open when ER Ca^2+^ stores are decreased from resting levels around 700 µM to about 200 µM (Alvarez and Montero, 2002; Villalobos et al., 2017). This partial depletion is sensed by the ER membrane-located Stromal Interaction Molecule 1 (STIM1) (Liou et al., 2005), when Ca^2+^ ions dissociate from its low affinity EF-Ca^2+^ binding domain. The decrease in ER-Ca^2+^ levels promotes STIM1 oligomerization and the interaction with the PM-localized calcium channel ORAI1 and/or TRP channels (Feske et al., 2006; Fahrner et al., 2017; Hernández-Morales et al., 2017; Secondo et al., 2018). The STIM1–ORAI1 interaction opens calcium release activated channels (CRAC), thus facilitating the calcium influx and the ER Ca^2+^-stores refilling for subsequent stimuli (Villalobos et al., 2017). In mammals, ORAI1 channel has two other isoforms, ORAI2 and ORAI3 encoded by homologous genes (Feske et al., 2006) and STIM1 only one STIM2 (Putney, 2018). In addition, there are two variants of ORAI1, the longer ORAI1α and the shorter ORAI1β, due to different translation points, with different membrane diffusion coefficients (Fukushima et al., 2012). The three ORAI isoforms can be activated by STIM1 with some variations in the pharmacology and biophysical properties of the channel (Lis et al., 2007; DeHaven et al., 2008).

Ca^2+^ release mechanisms in the ER are mediated by two major Ca^2+^ permeable channels: (1) ryanodine receptors (RyRs) (Fleischer et al., 1985) and (2) inositol trisphosphate (IP_3_) receptors (IP_3_Rs) (Berridge, 1983; Streb et al., 1983) (**Figure 1**). RyRs open mainly when intracellular Ca^2+^ rises, thus acting as a ligand in a mechanism called, Ca^2+^-induced Ca^2+^ release (CICR) (Endo et al., 1970). ER-Ca^2+^ release by IP_3_Rs starts at the PM, where agonist-induced G-protein-coupled receptors (GPCRs) activates the phospholipase C (PLC) enzyme. The PLC selectively induces the hydrolysis of the phosphatidylinositol 4,5-bisphosphate (PIP2), releasing diacylglycerol (DAG) and the second messenger IP_3_, which diffuses to the ER activating its receptor (Berridge, 1987).

In mitochondria, the principal calcium channel is the mitochondrial calcium uniporter (MCU), a Ca^2+^-activated, calcium channel, whose molecular machinery has been recently reported (Perocchi et al., 2010; Baughman et al., 2011; Sancak et al., 2013). MCU opens when large [Ca^2+^] microdomains develop at the mouth of this Ca^2+^-operated, calcium channel. The huge mitochondrial potential (ΔΨ), close to −180 mV, promotes the Ca^2+^ removal from cytosol into mitochondria (Valero et al., 2008; Villalobos et al., 2017), clearing large cytosolic Ca^2+^ loads formed during VGCCs opening in excitable cells (Villalobos et al., 2001) or during Ca^2+^ release from the ER (Rizzuto et al., 1998). In various cellular types, SOCE regulation by mitochondria depends on the Ca^2+^ buffering capability of these organelles, being able to prevent the slow, Ca^2+^-dependent inactivation of Ca^2+^ release activated calcium channels (CRAC) (Gilabert, 2000; Hernández-Morales et al., 2017).

## Calcium Permeable Channels In Cancer

### Voltage-Gated Calcium Channels

Recent data provide evidence of the importance of the calcium permeable channels in cancer. Regarding VGCCs, altered gene expression profiles has been related with different cancer types (Wang et al., 2015a) and it has been proved that different calcium channels blockers prevent cancer invasion (Buchanan and McCloskey, 2016; Jacquemet et al., 2016).

Experimental and bioinformatic analysis showed that different subunits of VGCCs such as Ca_V_1.2, Ca_V_1.3, Ca_V_2.2, Ca_V_3.1, Cav3.2 and Ca_V_3.3 are involved in the development and progression of different types of cancer, showing a significantly overexpression in breast cancer. Ca_V_1.4 only showed up-regulation in testis cancer, whereas Ca_V_2.1, Ca_V_1.2 and Ca_V_1.3 were deeply overexpressed in a wide range of cancer types (Wang et al., 2015a). More specifically, the high voltage-activated Ca_V_1.2 channel, commonly localized in cardiac, skeletal, smooth muscle, neurons, fibroblasts and pancreatic cells (Lipscombe et al., 2004; Tajada et al., 2013; Dixon et al., 2015), is overexpressed in most cancer types, including colorectal, gastric, leukemia, brain, uterus, breast, pancreatic, sarcoma, skin and prostate (Wang et al., 2015a). The Ca_V_1.3 is expressed in most of the same cells that express Ca_V_1.2, as well as, neuroendocrine, amacrine cells, auditory hair cells, photoreceptors and the sinoatrial node where it contributes to pacemaking (Platzer et al., 2000; Lipscombe et al., 2004; Moreno et al., 2016). The Ca_V_1.3 is highly expressed in most types of cancer, including breast and prostate cancer, but also in brain, colorectal, gastric, bladder, lung, esophageal and uterine tumors (Chen et al., 2014; Wang et al., 2015a; Buchanan and McCloskey, 2016; Jacquemet et al., 2016). See **Table 1** for a summary of calcium permeable channels involved in different cancer conditions. Furthermore, the carboxy terminus cleavage of both Cav1.2 and Cav1.3 promotes the altered expression of different proteins such as TRPV4 and small conductance potassium channels (SK3 and SK2) through the CCAT transcription factor or by direct translocation to the nucleus (Gomez-Ospina et al., 2006; Gomez-Ospina et al., 2013; Lu et al., 2015). Hence, additionally to the Ca^2+^ influx cancer-related signaling, both c-terminus can contribute to cancer hallmarks by modulation of other channels. It is also important to highlight the number of protein interaction sites described in the VGCCs, where different proteins modulate transcription factors such as CREB, NFAT and calmodulin, consequently linking ion channels with transcription factors involved in cancer progression (Mancini and Toker, 2009; Xiao et al., 2010; Berchtold and Villalobo, 2014).

**Table 1:** Calcium permeable channels involved in different cancer types.

Ca^2+^ permeable channels		Cancer types	Expression profile	Reference
VGCCs	Ca_V_1.2	Brain, breast, colorectal, pancreas, prostate, uterus, skin and esophageal	Gene upregulation	(Kale et al., 2015; Wang et al., 2015a; Cui et al., 2017; Anderson et al., 2019)
	Ca_V_1.3	Breast, prostate, colorectal, bladder, gastric, uterus, lung, brain and esophageal	Gene and protein upregulation and gene downregulation	(Chen et al., 2014; Wang et al., 2015a; Phan et al., 2017; Prevarskaya et al., 2018)
	Ca_V_1.4	Testis	Gene upregulation	(Wang et al., 2015a)
	Ca_V_2.1	Leukemia, sarcoma, ovarian, brain, uterus, lung, cervix, colorectal, esophageal and gastric	Gene upregulation and downregulation	(Hao et al., 2006; Sabates-Bellver et al., 2007; Wang et al., 2015a; Phan et al., 2017)
	Ca_V_2.2	Breast, brain and prostate	Gene up and downregulation	(Sun et al., 2006; Grasso et al., 2012; Wang et al., 2015a; Phan et al., 2017)
	Ca_V_3.1	Breast, glioma, lung, prostate, colorectal, pancreas and gastric	Gene upregulation and downregulation	(Latour et al., 2004; Wang et al., 2015a; Cui et al., 2017; Anderson et al., 2019)
	Ca_V_3.2	Prostate, renal, gastric, ovarian, brain, breast, bladder, lung, colon and skin	Gene and protein up and downregulation	(Gackière et al., 2008; Cui et al., 2017; Phan et al., 2017)
	Ca_V_3.3	Breast, sarcoma, esophagus and gastric	Gene upregulation	(Wang et al., 2015a; Anderson et al., 2019)
TRPs	TRPC1	Breast, colorectal, esophageal, gastric and liver	Gene and protein upregulation	(Dhennin-Duthille et al., 2011; Villalobos et al., 2016; Cui et al., 2017; Anderson et al., 2019)
	TRPC3	Breast and ovarian	Gene and protein upregulation	(Aydar et al., 2009; Yang et al., 2009a; Tao et al., 2013)
	TRPC4	Renal	Gene downregulation	(Veliceasa et al., 2007)
	TRPC6	Breast, liver, stomach, prostate and glia	Gene and protein upregulation	(El Boustany et al., 2008; Guilbert et al., 2008; Aydar et al., 2009; Cai et al., 2009; Ding et al., 2010; Prevarskaya et al., 2018; Anderson et al., 2019)
	TRPM6	Colorectal	Gene downregulation	(Xie et al., 2018; Anderson et al., 2019)
	TRPM7	Breast, pancreas, ovarian, gastric and colorectal	Gene and protein upregulation	(Hoshino et al., 1988; Kim et al., 2008; Middelbeek et al., 2012; Rybarczyk et al., 2012; Wang et al., 2014; Bose et al., 2015; Anderson et al., 2019)
	TRPM8	Pancreas, prostate, bladder, skin, breast, colorectal, and lung	Gene and protein upregulation and gene downregulation	(Schmidt et al., 2006; Prevarskaya et al., 2007; Chodon et al., 2010; Yee et al., 2010; Liu et al., 2014; Cui et al., 2017; Anderson et al., 2019)
	TRPV4	Skin, renal and bladder	Gene and protein downregulation	(Fusi et al., 2014; Mizuno et al., 2014; Cui et al., 2017; Prevarskaya et al., 2018)
	TRPV6	Breast, prostate, thyroid, colon, lung and ovary	Gene and protein upregulation and gene downregulation	(Wissenbach et al., 2001; Zhuang et al., 2002; Bolanz et al., 2008; Dhennin-Duthille et al., 2011; Fan et al., 2014; Cui et al., 2017)
SOCs	ORAI1	Glia, breast, skin, colorectal, pancreas, esophageal, lung and renal	Gene and protein upregulation	(McAndrew et al., 2011; Motiani et al., 2013a; Kim et al., 2014; Zhu et al., 2014b; Cui et al., 2017; Anderson et al., 2019)
	ORAI3	Prostate, breast and lung	Gene and protein upregulation and gene downregulation	(Abeele et al., 2002; Ay et al., 2013; Motiani et al., 2013b; Dubois et al., 2014; Villalobos et al., 2016; Cui et al., 2017)
	STIM1	Glia, cervix, breast, lung, skin, liver, pancreas and colorectal	Gene and protein upregulation	(Scrideli et al., 2008; Chen et al., 2011; McAndrew et al., 2011; Li et al., 2013; Yang et al., 2013; Umemura et al., 2014; Wang et al., 2015b; Wang et al., 2016; Cui et al., 2017; Anderson et al., 2019)
	STIM2	Colorectal, skin, breast and glia	Gene upregulation and protein downregulation	(Ruano et al., 2006; Aytes et al., 2012; Sobradillo et al., 2014; Stanisz et al., 2014; Cui et al., 2017; Anderson et al., 2019)

Moreover, compelling studies point to the emerging role of VGCCs accessory subunits in cancer (Haworth and Brackenbury, 2019). The VGCCs are associated with these non-pore forming subunits that tune the biophysical properties of the channel, including activation, gating kinetics or trafficking to the PM (Dolphin, 2016). The increased expression of the Ca_V_1.x and the Ca_V_2.x accessory subunits, α_2_δ and β, have been related with different cancer hallmarks in liver, ovary, prostate, pancreas, lung, and colon tumors (Mitra et al., 2011; Cromer et al., 2015; Warnier et al., 2015; Yu et al., 2016; Badr et al., 2018; Gao et al., 2018). The involvement of these subunits in cancer adds an extra complexity to the VGCCs- cancer link mechanism.

### TRP Calcium Permeable Channels

The Ca^2+^ permeable members of the TRP channel superfamily, which have a high diversity of gating mechanism (Nilius and Owsianik, 2010; Gees et al., 2012), are differently regulated in some cancer types. The remodeling of Ca^2+^ entrance and release pathways favors the phenotypic switch from normal to high proliferative state. For example, the expression of several TRP channels is elevated in different common carcinomas, e.g. TRPC1 in breast cancer (Dhennin-Duthille et al., 2011), TRPC3 in some breast and ovarian tumors (Aydar et al., 2009; Yang et al., 2009a; Tao et al., 2013), TRPC6 in breast, liver, stomach and glia cancers (El Boustany et al., 2008; Aydar et al., 2009; Cai et al., 2009; Ding et al., 2010; Dhennin-Duthille et al., 2011; Wen et al., 2016; Diez-Bello et al., 2019), TRPM7 in breast, pancreas, ovarian and gastric cancers (Hoshino et al., 1988; Kim et al., 2008; Kim et al., 2012; Middelbeek et al., 2012; Rybarczyk et al., 2012; Wang et al., 2014; Yee et al., 2015). TRPM8 is highly expressed at both mRNA and protein levels in tumor cells from different tissues: breast, pancreas, prostate, colorectal and lung (Schmidt et al., 2006; Chodon et al., 2010; Yee et al., 2010; Liu et al., 2014), but the expression is decreased during metastasis in prostate cancer cells via TCAF1 associated factor (Gkika et al., 2015). In this regard, a new pore-independent mechanism has been revealed, where TRPM8 inhibit endothelial cell migration via Rap1, a small GTPase, protein-protein interaction (Genova et al., 2017). TRPV6 expression levels are significantly high in different human carcinomas tissues, such as, breast, prostate, thyroid, colon and ovary (Wissenbach et al., 2001; Zhuang et al., 2002; Bolanz et al., 2008; Dhennin-Duthille et al., 2011). In contrast, the expression of other TRP channels appears to be decreased in cancer. For instance, TRPC4 channels in renal carcinoma (Veliceasa et al., 2007), TRPM6 in colorectal tumors (Xie et al., 2018), TRPV4 in skin and bladder tumor cells (Fusi et al., 2014; Mizuno et al., 2014; Yu et al., 2019) or the TRPV6 in lung carcinomas (Fan et al., 2014). In conclusion, the TRP channels superfamily expression levels have been described to be either up or down regulated in different tumors (**Table 1**). The specific molecular mechanism of these channels in cancer is not fully understood, but recent evidence suggests a key role for some of these channels in the cancer hallmarks (Villalobos et al., 2017; Prevarskaya et al., 2018). In addition, some members of the TRPC subfamily contribute to SOCE forming channel complexes with STIM1 and ORAI1 (Molnár et al., 2016; Villalobos et al., 2016). This set of proteins does not associate to form heterohexamers ORAI1/TRPC1/STIM1 complexes. Instead, ORAI1 and TRPC1 subunits form distinctive pore structures each responsible for its calcium selective permeability (Cheng et al., 2013). Further, TRPC1 activation is mediated by STIM1 binding, but TRPC1 trafficking to the membrane depends on ORAI1-based Ca^2+^ entry. Therefore, ORAI1/STIM1 and TRPC1/STIM1 assemble into two separate channels providing individual calcium signals for regulation of different cell functions (Ambudkar et al., 2017; Villalobos et al., 2018). In fact, there is a long-term controversy on the function of TRPC channels in SOCE. The final consensus is that they may play a role in some cell types but not in others (Ambudkar et al., 2017). In anterior pituitary cells from mice lacking either ORAI1 or the seven TRPC1–7 channels, it was recently shown that SOCE required only ORAI1 channels but not any of the TRPC channels (Núñez et al., 2019). In human colonic cells, it seems that SOCE is mediated only by ORAI1 channels in normal cells. However, in colon cancer cells, SOCE depends on both ORAI1 and TRPC1 channels (Sobradillo et al., 2014) as reviewed next. Other member of the TRP channel superfamily, which is translocated to the plasma membrane via ORIA1-mediated mechanism, is the TRPV6 channel controlling the cancer cell survival (Raphaël et al., 2014).

### Store Operated Calcium Channels

The mechanisms through SOCCs members contribute to cancer progression differ according to the molecular entity of the protein and the cancer type-specificity. It is important to remember that SOCCs are not just a single molecular unit, but rather multiprotein complex which includes PM and ER-membrane components, ORAI and STIM proteins respectively. Although calcium signal remodeling involves changes of different Ca^2+^-permeable channels, Ca^2+^ entry through ORAI channels is particularly important, as its dysregulation contribute to cancer features such as sustained proliferation, apoptosis resistance and invasion.

ORAI1 key component of SOCE, is essential for human brain tumor glioblastoma invasion. In these cells, the ORAI1 expression is upregulated causing an increased ORAI1-based calcium influx (Motiani et al., 2013a). In this regard, some breast cancer cell lines exhibit augmented levels of ORAI1, SOCE and the remodeling of the calcium influx associated with invasive stimuli (McAndrew et al., 2011; Zhu et al., 2014b). The ORAI1 involvement occurs also in melanoma cells, playing a central role in cell migration and proliferation, most likely via ERK signaling (Umemura et al., 2014). Furthermore, the ORAI1 interaction with SK3, a small conductance calcium-activated potassium channel, has been correlated with invasion and metastasis in colon and breast cancer, respectively, via SigmaR1 chaperone (Gueguinou et al., 2017). In renal carcinomas, the ORAI1 expression levels in cancer tissues were statistically higher than the levels in the adjacent normal parenchymal tissues, promoting cell proliferation and migration (Kim et al., 2014). These results suggest that, regardless the feasible differences in the cancer type-specificity and mechanism, ORAI1 plays a pivotal role in proliferation and invasion. However, this assumption is controversial because other authors propose that proliferation and apoptosis resistance is due to the ORAI3 remodeling. For instance, in prostate cancer cells, the overexpression of ORAI3 promotes two cancer hallmarks at once, enhanced proliferation and apoptosis resistance (Dubois et al., 2014). The ORAI3 increased expression levels favors the formation of ORAI1-ORAI3 heterohexamers over the ORAI1 homohexamers. The shift in the ORAI1/ORAI3 ratio reduces the Ca^2+^ influx from the extracellular space, promoting the enhanced apoptosis resistance shown by these cells (Abeele et al., 2002). These results highlight the essential role of ORAI1 in death related process (Flourakis et al., 2010). The downregulation of the ORAI1-based SOCE is associated with increased store independent calcium entry (SICE), promoting cancer cells proliferation via the Ca^2+^/calcineurin-dependent transcription factor (Dubois et al., 2014). The ORAI3 expression is also elevated in breast cancer cells, where enhanced ORAI3-dependent SOCE result in apoptosis resistance, proliferation, and invasion in an estrogen receptor-dependent way (Motiani et al., 2013b). Association between elevated ORAI3 expression, enhanced ORAI3-based SOCE and high proliferation levels was also reported for non-small-cell lung adenocarcinoma (Ay et al., 2013) (**Table 1**).

The Ca^2+^ sensor STIM1 is overexpressed in different carcinomas, e.g., glioblastoma, cervix, breast, lung, liver, melanoma and colon (Scrideli et al., 2008; Chen et al., 2011; McAndrew et al., 2011; Li et al., 2013; Yang et al., 2013; Umemura et al., 2014; Wang et al., 2015b; Wang et al., 2016). In fact, STIM1 expression level is enhanced in early-stage cervical cancers, and using STIM1 siRNA, both SOCE and proliferation were inhibited in the cervical cancer cells. Furthermore, there is a positive correlation of local migration, tumor size and angiogenesis with STIM1 levels in cervical cancers cells (Chen et al., 2011). The abolition of STIM1 also prevents the cell proliferation and induces G0/G1 phase arrest of human glioblastoma cells (Li et al., 2013). In alveolar epithelial cells, STIM1 silencing attenuates the tumor growth, arresting cells in G1 phase in a p21 and cyclin D1-mediated pathway (Wang et al., 2016). In the same direction, STIM1 siRNA inhibits the migration and invasion abilities of the highly invasive tumor hepatic cells (Yang et al., 2013). Increased levels of STIM1 promotes growth and migration in colorectal cancer cells (CRC) and STIM1 elevated expression in CRC patients is associated with tumor size, invasion and metastasis (Wang et al., 2015b). Moreover, microarray analysis data show that, the basal-breast cancer, a breast cancer subtype with a very dismal prognosis, is correlated with an altered relationship between the ORAI1 activators STIM1 and STIM2, characterized by high STIM1/STIM2 ratios (McAndrew et al., 2011; Berna-Erro et al., 2017) (**Table 1**).

STIM1 homolog STIM2 senses smaller changes in ER-Ca^2+^ concentrations than STIM1, suggesting different cellular functions for these two Ca^2+^ sensors (Brandman et al., 2007). STIM1 detects deeper ER-Ca^2+^ depletions, due to its higher affinity for calcium, thus enabling the refilling of the depleted stores, while STIM2 detects lighter ER-Ca^+2^ depletions, keeping ER-Ca^2+^ concentrations within tight limits (Berna-Erro et al., 2017). Elevated STIM2 expression levels have been found in different cancer types, colorectal cancer (Aytes et al., 2012), human melanoma (Stanisz et al., 2014) and glioblastoma multiform tumors (Ruano et al., 2006). In colorectal cancer cells the high STIM2 expression levels has been related with a reduced invasive phenotype, suggesting a cancer cell growth suppressor function in contrast to STIM1 (Aytes et al., 2012; Berna-Erro et al., 2017). In the same vein, STIM2 overexpression was detected in human melanoma, where STIM2 siRNA treatment produced enhanced proliferation (Stanisz et al., 2014). These results correlate with the idea that, low STIM1/STIM2 ratios may be associated with less aggressive tumor conditions.

It is noteworthy that, while STIM2 gene expression levels are increased in CRC cells, STIM2 protein is practically absent in tumor cells, leading to the partial depletion of calcium stores (Sobradillo et al., 2014; Villalobos et al., 2016). The STIM2 loss in tumor cells moves internal Ca^2+^ store concentration near to the SOCE activation threshold and promotes apoptosis resistance and cell survival (Sobradillo et al., 2014). These discrepant results reveal the complexity role of STIM proteins in cancer and expand the classical function of controlling intracellular Ca^2+^ homeostasis. Additionally, the recent discovery of new STIM2 mRNA isoforms with opposite effects, might clarify these apparently contradictory reports. In these new reports, the novel variant STIM2.1 inhibits SOCE, whereas the former isoform STIM2.2 promotes it (Miederer et al., 2015) as detailed in (Berna-Erro et al., 2017).

## Intracelular Calcium Homeostasis

Intracellular Ca^2+^ concentrations control many different physiological and cellular functions at global and local levels, such as gene transcription in the nucleus, cell respiration and ATP synthesis in mitochondria or exocytosis in plasma membrane. To regulate this wide range of physiological Ca^2+^-dependent processes, the cells maintain Ca^2+^ concentrations tightly controlled. This regulation is known as calcium homeostasis.

To keep calcium homeostasis and considering that Ca^2+^ is a cation and does not cross freely cell membranes, Ca^2+^ is transported through PM and internal membranes by particular transmembrane proteins, including Ca^2+^ permeable channels, Ca^2+^ pumps and ion transporters (Bong and Monteith, 2018; Villalobos et al., 2018). As we mentioned above, Ca^2+^ permeable channels enable Ca^2+^ entry into cells forced by the electrochemical gradient, formed by the difference in Ca^2+^ concentration between the internal (100 nM) and extracellular (1 mM) space and the membrane potential of about −60 mV. The calcium entry rises intracellular [Ca^2+^] triggering different cell process. Then, it is removed from the cytosol and transported back to the extracellular medium or uptake into the intracellular Ca^2+^ stores by Ca^2+^ pumps and transporters, restoring the basal Ca^2+^ levels (Villalobos et al., 2018) (**Figure 1**).

This removing-Ca^2+^ process requires energy provided by the ATP hydrolysis for Ca^2+^-pumps or the energy stored in the electrochemical gradient of other cation for transporters. For example, the Na^+^/Ca^2+^ exchanger transports three Na^+^ ions down its gradient in exchange of one Ca^2+^ cation against its electrochemical gradient (Reuter and Seitz, 1968). These dynamic Ca^2+^ signals differ with regard to their mechanisms of generation, spatial distributions and temporal properties, but are coordinated in space and time for a specific purpose. These calcium signals are characterized in different cell types and include calcium waves (Berridge et al., 2003; Parkash and Asotra, 2010), sparks (Cheng et al., 1993), calcium puffs (Yao et al., 1995), spikes (Meyer and Stryer, 1991), flickers (Wei et al., 2009), sparklets (Wang et al., 2001; Navedo et al., 2005; Tajada et al., 2017), etc. Compelling evidence show that variations in cellular Ca^2+^ dynamics participate in the normal and pathological signal transduction that controls cell proliferation and survival (Prevarskaya et al., 2018). Abnormal remodeling in Ca^2+^ homeostasis leads to the disruption of Ca^2+^ signaling resulting in a wide range of pathological conditions such as cell migration, excessive proliferation, invasion and programmed cell death resistance (Prevarskaya et al., 2011; Monteith et al., 2012; Prevarskaya et al., 2014).

Intracellular Ca^2+^-storage organelles like the ER, mitochondria and the recently emerged as a major intracellular Ca^2+^ storage, lysosomes (Lloyd-Evans and Waller-Evans, 2019), also hold their own Ca^2+^ transporters. The ER is the largest single organelle and the most important intracellular Ca^2+^ store (Lam and Galione, 2013). It operates as a dynamic Ca^2+^ store thanks to the combined action of Ca^2+^ channels and transporters in the ER-membrane, and the Ca^2+^-binding proteins in the ER-lumen, working as a high-capacity Ca^2+^ buffering system (Verkhratsky, 2005). Ca^2+^ is continuously leaking from the ER through leak channels still not identified. Nevertheless, Ca^2+^ concentration within the ER remains high due to the continuous activity of the endoplasmic reticulum Ca^2+^-ATPases (SERCA) that replenish Ca^2+^ stores (MacLennan, 1970) (**Figure 1**). On the other hand, activation of the IP_3_Rs, receptor-operated Ca^2+^ channels, release Ca^2+^ from intracellular store sites, contributing to the maintenance of a total amount of Ca^2+^ in the lumen similar to the extracellular space, in the mM range (Alvarez and Montero, 2002; Foskett et al., 2007). Because the lumen contains high concentrations of Ca^+2^ binding proteins (calreticulin, calsequestrin, annexin, calnexin, etc.) the concentration of free Ca^+2^ has been estimated to be between 100 and 700 µM (Foskett et al., 2007; Yáñez et al., 2012).

In the mitochondria, the Ca^2+^ uptake is conducted by the MCU and the Na^+^/Ca^2+^ (NCLX) and Ca^2+^/H^+^ (HCX) exchangers are responsible for the mitochondrial Ca^2+^ release in exchange for Na^+^ and H^+^, respectively (Bononi et al., 2012; Srinivasan et al., 2015) (**Figure 1**). A large increase in Ca^2+^, located near enough the MCU, triggers the influx of calcium to the mitochondrial matrix, rising the mitochondrial [Ca^2+^] to very high concentrations near the mM level (Pozzan et al., 2000; Contreras et al., 2010). Mitochondria are also in dynamic contact with other organelles including the ER. These transient contacts between ER and mitochondria are essential to generate the highly localized and concentrated Ca^2+^ microdomains, facilitating the Ca^2+^ transport into mitochondria (Paupe and Prudent, 2018). Through this mitochondrial-ER crosstalk, IP_3_Rs control the mitochondrial Ca^2+^ uptake and therefore metabolism and cell destiny (Cui et al., 2017). Although the mitochondria play an important role in Ca^2+^ homeostasis, the “*in vivo*” buffering capacity of this intracellular organelle is not particularly relevant (Pozzan et al., 2000).

Mitochondria not only modulate cytosolic Ca^2+^ signal. Mitochondrial calcium is also involved in the control of metabolism, ATP production and the regulation of cell death. Mitochondrial calcium regulates three important enzymes of the Krebs cycle: ketoglutarate, isocitrate, and pyruvate dehydrogenase. The effect of mitochondrial Ca^2+^ accumulation in Krebs cycle is the increase in the respiration rate and ATP synthesis (Mammucari et al., 2018). On the other hand, an excess of mitochondrial Ca^2+^ uptake, known as “mitochondrial calcium overload” triggers mitochondrial permeability transition pore (mPTP) opening. This is a high-conductance channel mediating mitochondrial swelling (Petronilli et al., 1993). This, together with other factors, triggers mitochondrial permeability transition, disruption of the mitochondrial potential and a massive release of cytochrome c, leading to the apoptosome activation and apoptosis (Giorgi et al., 2012; Mammucari et al., 2018).

The plasmalemma also displays many different calcium channels, ATP pumps and transporters to maintain the intracellular Ca^2+^ homeostasis. We have mentioned above the channels responsible of the Ca^2+^ influx. Now we will briefly describe the players involved in the transport of calcium out of the cell to maintain the Ca^2+^ electrochemical gradient. Two players in the Ca^2+^ outflow from cells have been described, the Na^+^/Ca^2+^ exchange transporter (NCX) and the plasma membrane Ca^2+^-ATPase (PMCA) (**Figure 1**). The NCX is in charge of Ca^2+^ extrusion from the cytoplasm against its electrochemical gradient without ATP consumption, in fact, the electrochemical gradient of sodium stores that energy. In addition, NCX can operate in reverse mode contributing to Ca^2+^ entry in different conditions, such as cellular activation (Blaustein and Lederer, 1999). The PMCA pump with a 1:1 Ca^2+^/ATP stoichiometry possesses a high-affinity but a low-capacity for Ca^2+^ transport, in total opposition to NCX (Noble and Herchuelz, 2007). This characteristic has conditioned the roles of these Ca^2+^ transporters. The PMCA performs a housekeeping role maintaining cytosolic Ca^2+^ around its basal level, whereas the NCX eliminates significant rises in intracellular Ca^2+^, but this model has been revised during the latest years. See (Brini and Carafoli, 2011) for review.

## Calcium Permeable Channel Modulators and Cancer

### Voltage-Gated Ca^2+^ Channel Modulators and Cancer

Several epidemiological investigations report that, calcium channel blockers used for the medical therapy of other pathologies are related with a reduced prostate cancer risk (Debes et al., 2004; Rodriguez et al., 2009), or at least a significantly tumor aggressiveness reduction (Poch et al., 2013). There is more discrepancy in studies of other cancer types, ranging from significant diminution in breast cancer risk (Fitzpatrick et al., 1997; Taylor et al., 2008) to no association between VGCCs blockers and cancer in colorectal, lung and breast tumors (Michels et al., 1998; Boudreau et al., 2008; Devore et al., 2015; Brasky et al., 2017; Bowles et al., 2019), including higher lung cancer risk correlated with exposure to calcium channels blockers (Rotshild et al., 2018).

Although, the potential benefit of the VGCCs blockers needs further preclinical studies, specific VGCCs antagonists, already approved by the FDA and EMA and used in clinical treatments, may be interesting for cancer treatments where individual VGCCs are overexpressed.

### CRAC Channel Modulators and Cancer

The identification of CRAC channels modulators has been an important challenge for researchers and pharmaceuticals, since their discovery in 1992 (Hoth and Penner, 1992), because they have been implicated in numerous human pathologies, including pancreatitis, immunodeficiency, occlusive vascular diseases and cancer (Gerasimenko et al., 2013; Tian et al., 2016; Stauderman, 2018).

Numerous compounds have been described as inhibitors of SOCE or CRAC channels, which can be classified into four groups: inorganics, aptamers, antibodies and organic molecules (Stauderman, 2018). The inorganic lanthanides, specifically the trivalent cations, Lanthanum (La^+3^) and Gadolinium (Gd^+3^) are potent but non-selective blockers of the CRAC current (Parekh and Putney, 2005). Both can block directly the CRAC pore formed by the loopI-II of ORAI1, but also block other Ca^2+^ channels and pumps, such as VGCCs, TRPs and PMCA (Trebak et al., 2002; Yeromin et al., 2006; Cui et al., 2017). However, Gd^3+^ is normally used in Ca^2+^ imaging experiments to discriminate endogenous SOCs from other Ca^2+^ permeable channels such as recombinant TRPs, because the concentration that efficiently blocks the endogenous pathway does not interfere with the TRPs (Trebak et al., 2002; Parekh and Putney, 2005). In addition, these chemical elements are not viable for therapeutic treatment due to toxicological concerns. Aptamer Y1, an artificial single-stranded oligonucleotide engineered to bind with high affinity the first extracellular domain of the ORAI1 protein, block SOCE in the nM range in mast cells (Sun et al., 2016). However, the oligonucleotides present a disadvantage to be consider a potential drug candidate, the administration route is restricted to intravenous, subcutaneous or local delivery, as happened with the antibodies (Stauderman, 2018). Among antibodies, 10F8 (Cox et al., 2013), Anti-CRACM1-IgG (Liu et al., 2017), hHG1/LG1 (Komai et al., 2017) and mAb 2C1.1 (Lin et al., 2013) have been reported to partial block SOCE or CRAC currents due to the low specificity against all ORAI isoforms. Considering the organic small agents, the 2-aminoethoxydiphenylborane (2-APB), a membrane permeable antagonist of the IP_3_-receptor activity, is widely used is SOCE experiments (Ma et al., 2000). Some studies reported that 2-APB inhibit cell proliferation and death resistance in gastric cancer cells (Sakakura et al., 2003; Cui et al., 2017) and cell migration in CRC cells (Abeele et al., 2002). Although, 2-APB is used as a reliable SOCE inhibitor (Bootman et al., 2002), it presents diverse effects on SOCE or CRAC currents. 2-APB is able to both, inhibit CRAC current without interference in the STIM1 and ORAI1 interaction (Yamashita et al., 2011) and directly activate ORAI3 channel independently of Ca^2+^ store depletion or STIM1 protein (Schindl et al., 2008). In addition, it presents a dose-dependent bimodal effect, stimulating CRAC currents at low concentration (less than 10 μM) and a transient increase followed by inhibition at higher (up to 50 μM) concentrations (Prakriya and Lewis, 2001). For this reason and because 2-APB modulates other proteins including SERCA (Missiaen et al., 2001), MagNum (Hermosura et al., 2002) and K^+^ channels (Wang et al., 2002), mitochondrial Ca^2+^ efflux (Prakriya and Lewis, 2001), gap junctions (Tao and Harris, 2007) and different TRP channels (Parekh and Putney, 2005; Derler et al., 2008), it should not be considered as a SOCE specific inhibitor (Parekh and Putney, 2005; Várnai et al., 2009). However, novel 2-APB derived compounds such as DBB-162AB and DPB-163AE with higher efficiency in SOCE inhibition and without unexpected interactions, are promising chemotherapeutic drugs (Goto et al., 2010).

Another class of small-molecules such as AnCoA4, ML-9, SB01990, KM06293 and RH01882 has been recently identified as SOCE inhibitors via different mechanism. AnCoA4 reduces the ORAI1 recruitment into puncta (Sadaghiani et al., 2014), ML-9 prevents STIM1 migration to ER-PM junctions (Smyth et al., 2008), SB01990, KM06293 and RH01882 modify the ORAI1 pore geometry, altering the Ca^2+^ selectivity and thereby reducing SOCE (Sampath and Sankaranarayanan, 2016; Cui et al., 2017).

Another compounds, imidazol, tenidap and SFK-96365 all classified as receptor-mediated Ca^2+^ entry blockers, were reported to inhibit CRAC currents in rat mast cells, but not in a specific manner (Franzius et al., 1994). For example, similar SKF96365 concentration used to block the CRAC currents, inhibits TRPC and TRPM channels with a comparable efficacy. The SKF-96365 was the first ORAI1 blocker used in cancer studies, specifically breast cancer. This imidazol derivative reduced tumor growth and invasion *in vivo* and inhibited cellular migration *in vitro* in breast cancer cells (Yang et al., 2009b). In other study, SKF-96365 significantly reduced tumor growth in esophageal cancer cells from immune deficient mouse, blocking ORAI-mediated SOCE and Ca^2+^ oscillations (Zhu et al., 2014a). Other compound targeting SOCE is the biomolecule Ohmline, a Edelfosine mimetic, that inhibits breast and colon cancer cells migration through the TRPC1/ORAI1/SK3 complex dissociation (Guéguinou et al., 2016).

Pyrazoles, heterocyclic organic compounds, specifically BTP1, BTP2 and BTP3, were identified by Abott and Astellas as SOCE blockers in Jurcat cells, using transcriptional and Ca^2+^ imaging assays (Djuric et al., 2000; Ishikawa et al., 2003; Stauderman, 2018). BTP2 (also known as Pyr2 or YM58483) is a pyrazole analogue that potently inhibits both TRPC-mediated and CRAC Ca^2+^ influx (He et al., 2005; Stauderman, 2018). However, similar to 2-APB and imidazole these compounds are not specific because they are involved in many other transport mechanisms. For instance, BTP2 also produces a PM depolarization via TRPM4 activation, therefore decreasing the Ca^2+^ driving force and consequently the Ca^2+^ influx inhibition (Takezawa et al., 2006). In contrast, other pyrazol-based compounds such as GSK-7975A (Derler et al., 2013), RO2959 (Chen et al., 2013) and the carboxamide GSK1349571A (Di Sabatino et al., 2009) seem to have reasonable selectivity with no affinity toward a wide range of receptors and ion channels and without affect TRPC1/5-mediated SOCE. Recently developed pyrazole analogues, such as, pyrazole-4-carboxamide (YW2065) and pyrimidine-2(1H)-thione derivative, have reported good anticancer activity in colorectal cancer cell lines compared to the standard drugs like sorafenib or oxaliplatin (Fahmy et al., 2016; Yang et al., 2019). More detailed information about the pharmacology of various CRAC channels modulators is revised in recent reports (Sweeney et al., 2009; Jairaman and Prakriya, 2013; Pevarello et al., 2014; Tian et al., 2016).

Other line of research is using small molecules compounds that modify the unbalance Ca^2+^ homeostasis in cancer cells. For example, the Nhr-BH4, a BCL-2 mimetic peptide that prevents the inhibition of the IP_3_R Ca_2+_ release and thus unblock the apoptosis resistance in breast cancer cells (Nougarede et al., 2018). The TAT-IDPs, other BCL-2 based peptide, potentiates the pro-apoptotic Ca^2+^ signaling in chronic lymphocytic leukemia cells avoiding the IP_3_R_2_ inhibition, an IP_3_R homolog, (Akl et al., 2013).

The importance of discovering new and specific inhibitors of CRAC channels and the development of new nanotechnology tools (Grolez et al., 2019), could be giant for the treatment of different human diseases. However, future pharmacology studies, and clinical trials will solve if these drugs are viable for therapeutic treatment.

## Aspirin, Nsaids and Polyamine Synthesis Blockers as Calcium Channel Modulators In Cancer

Overwhelming evidence provided in the last decades strongly suggest that acetylsalicylic acid (ASA), or aspirin provide protection against several forms of cancer, particularly those in the gastrointestinal tract (Chan et al., 2012; Rothwell et al., 2012; Drew et al., 2016). Same evidence has been also provided for a series of non-steroidal anti-inflammatory drugs (NSAIDs) like ibuprofen, flurbiprofen and sulindac, either alone or, in combination with other compounds like difluoromethylornithin (DFMO), a suicide inhibitor of polyamine biosynthesis (Tegeder et al., 2001; Rostom et al., 2007; Meyskens et al., 2008; Thompson et al., 2010; Chan et al., 2012; Dolejs et al., 2016). Evidence includes multiple data in cell lines and model animals, but also tens of epidemiological studies. Aspirin and NSAIDs inhibit several characteristic cancer cell hallmarks, including unrestrained cell proliferation, cell migration and invasion and cell death resistance. Numerous series of epidemiological evidences suggested that regular use of aspirin and/or several NSAIDs provide significant reduction in the risk of developing several forms of cancer and, particularly colorectal cancer. However, recommendation to use these over the counter drugs for cancer chemoprevention is hindered by the well-known secondary effects and/or risks of the regular use of NSAIDs leading to the conclusion that if, for any other reason or condition, individuals use NSAIDs regularly, they are probably protected against several forms of cancer. For any other situation, recommendation remains at hands of Doctors, that must evaluate whether the risk of taking NSAIDs is worthy in some cases of high risk of cancer. In fact, several studies have concluded that individuals at high risk of developing some forms of hereditary cancer like Lynch Syndrome may benefit of taking aspirin and/or NSAIDs to help protecting them from cancer (Burn et al., 2011; Yurgelun and Hampel, 2018). This may also apply to those patients that have received surgery for removing of polyps and/or tumors in the gastrointestinal tract that are also at high risk of recurrence. In these particular cases, the benefits are supported by several clinical trials that have addressed specifically the treatment and dose. For example, a clinical trial based in the combination of sulindac with DFMO in patients with previous tumors indicate that this combination may reduce nearly 90% the risk of developing recurrences (Meyskens et al., 2008; Sporn and Hong, 2008), revised in (Laukaitis and Gerner, 2011).

The action mechanisms of these drugs involved in preventing cancer cell hallmarks and cancer itself have been addressed in a large series of studies but they remain obscure. Most studies have addressed the contribution of inflammation to carcinogenesis (Balkwill and Mantovani, 2001; Coussens and Werb, 2002) and the benefits of inhibiting inflammatory mechanisms in cancer cell (Hudes et al., 2013; Wu et al., 2013). However, although inflammation definitely contributes to cancer risk, several evidences suggest that additional and/or alternative mechanisms may contribute as well and some relevant mechanisms include modulation of the expression and/or activity of calcium permeable channels in cancer cells (Déliot and Constantin, 2015; Hoth, 2016; Prevarskaya et al., 2018).

Núñez et al. (2006) reported that salicylic acid, the most important metabolite of aspirin, inhibited store-operated Ca^2+^ entry (SOCE) and SOCE-dependent cell proliferation in colon cancer cells (Núñez et al., 2006; Valero et al., 2008). This effect cannot be attributed to inhibition of cyclooxygenase (COX) as salicylic acid lacks the acetyl moiety involved in COX acetylation, the mechanism involved in COX inhibition by aspirin. Also, it is achieved at concentrations far lower than the necessary to inhibit COX expression. Finally, the effects are similar in cells lacking COX2 expression implying that an alternative target is involved. On the other hand, same results are obtained by other NSAIDs including ibuprofen, indomethacin, sulindac and R-flubiprofen, the enantiomer unable to inhibit COX activity. Accordingly, salicylate and other NSAIDs inhibit SOCE and cell proliferation independently of COX activity and/or gene expression. Since SOCE is critical for cell proliferation, these drugs inhibit cell proliferation more likely because they inhibit SOCE rather than inflammatory pathways. This paradoxical view could be reconciled taken into account that SOCE may also contribute to inflammatory pathways as it is key in the activation of all cell types, including inflammatory cells. Importantly, inhibition of cell proliferation by salicylic acid is counteracted simply by enhancing extracellular Ca^2+^ just to restore Ca^2+^ entry in colon cancer cells. When taken together, these data indicate that salicylate (aspirin) and NSAIDs prevent cell proliferation in tumor and non-tumoral cells by inhibiting SOCE in a COX-independent manner.

In search for the mechanism of inhibition of SOCE by salicylate and other NSAIDs, Villalobos and cols. tested the effects of these drugs directly on Ca^2+^-release activated currents (CRAC) responsible for SOCE in rat basophilic leukemia cells (RBL) and other cell types (Muñoz et al., 2011). These currents display a strong Ca^2+^-dependent inactivation (Hoth et al., 1997; Fierro and Parekh, 2000) that render them unable to operate in a few seconds unless nearby mitochondria take up this calcium and sustain the current. In this sense, CRAC channels are dictated not only by the filling state of intracellular Ca^2+^ stores but also by mitochondria (Villalobos et al., 2018). Inhibition of the MCU, the calcium channel responsible of mitochondrial Ca^2+^ uptake, with ruthenium salts derivatives like RU386 or mitochondrial uncouplers like FCCP, that collapse the mitochondrial potential, the driving force for mitochondrial Ca^2+^ uptake, inhibit mitochondrial ability to take up Ca^2+^, thus leading to Ca^2+^-dependent inactivation of CRAC channels (Villalobos et al., 2019). Similar results have been obtained by MCU downregulation (Samanta et al., 2019). Salicylate and other NSAIDs are carboxylic acids bound to aromatic rings, the typical chemical structure of mitochondrial uncouplers. Accordingly, these drugs are able to decrease the mitochondrial potential or even collapsing it depending on concentration (Muñoz et al., 2011; Scatena, 2012; Hernández-Morales et al., 2017). As a consequence, at low concentrations they work as mild mitochondrial uncouplers that prevent mitochondrial Ca^2+^ uptake, leading to the Ca^2+^-dependent inactivation of CRAC channels and SOCE inhibition in RBL cells. In conditions of high intracellular concentration of the Ca^2+^ buffer, when the Ca^2+^-dependent inactivation cannot be achieved regardless of mitochondria, neither salicylate, NSAIDs nor MCU removal promote CRAC inactivation or SOCE inhibition. Therefore, salicylate and selected NSAIDs do not inhibit CRAC channels directly. Instead, these drugs promote the Ca^2+^-dependent inactivation of these channels by preventing the Ca^2+^ uptake by mitochondria exactly as if we remove the MCU (Villalobos et al., 2019). These same results have been obtained also in other cell types as well, including vascular smooth muscle cells (VSMCs) (Muñoz et al., 2011; Muñoz et al., 2013) and colonic cells both normal and tumoral (Hernández-Morales et al., 2017).

Interestingly, the effects of salicylate and NSAIDs are achieved at very low concentrations in normal VSMCs leading to a full arrest of cell proliferation. VSMCs undergo phenotypic remodeling in different physiopathological situations. For example, upon damage, VSMCs may transform from differentiated, contractile cells to a proliferative and migrating phenotype that restores injury and returns to the differentiated phenotype. This process helps restoring damaged vessels but it may not be as beneficial if excessive proliferation occludes vessels as it happens in restenosis. This transdifferentation process is mediated by a change in the prevalence of different Ca^2+^-permeable channels. In the differentiated phenotype, voltage-gated channels sensitive to dihydropyridines are prevalent. However, in the proliferative state, store-operated Ca^2+^ channels take the lead. These channels show strong Ca^2+^-dependent inactivation that is prevented by mitochondria. Accordingly, low concentrations of salicylate and NSAIDs inhibit SOCE and cell proliferation in these cells during the proliferative phenotype but have no effect on Ca^2+^ entry during the differentiated phenotype. In fact, they have been proposed for treatment of restenosis, or excessive VSMC proliferation after stent implantation in coronary arteries (Weber et al., 2000; Koo et al., 2007; Dannoura et al., 2014).

As stated above, sensitivity of store-operated channels to aspirin and NSAIDs depends on mitochondria. As mitochondria from normal and tumor cells differ because of the Warburg effect, perhaps mitochondrial control of store-operated channels may be different as well in normal and colon cancer cells. Hernández-Morales et al. (2017) reported recently that this is indeed the case, at least in normal and colon cancer cells. Mitochondria from normal cells are not powerful enough to remove completely the Ca^2+^-dependent inactivation of store-operated channels. However, in cancer cells, probably because of the Warburg effect, mitochondria display enhanced mitochondrial potential and remove efficiently Ca^2+^, thus preventing efficiently the Ca^2+^-dependent inactivation of store-operated channels and sustaining Ca^2+^ entry (Hernández-Morales et al., 2017). Supporting this idea, the RNA expression analysis of genes involved in mitochondrial Ca^2+^ transport indicate that, MCU positive-modulator genes are overexpressed while negative-modulators are underexpressed in colon cancer cells (Pérez-Riesgo et al., 2017).

As stated above in the review, another difference between normal and colon cancer cells is that SOCE in normal colonic cells involves only ORAI1-mediated CRAC channels. In contrast, in colon cancer cells, SOCE is mediated by both ORAI1 and TRPC1 channels. Whereas ORAI1 channels in normal cells are essentially inactivating, in colon cancer cells, perhaps the involvement of TRPC1 channels may contribute to sustain currents. A possibility recently pointed out is that Na^+^ influx mediated by TRPC1 channels may favor activity of the mitochondrial Na^+^/Ca^2+^ exchanger, in turn preventing mitochondrial Ca^2+^ overload and the ensuing formation of reactive oxygen species (ROS) and ROS-dependent inactivation of ORAI1 channels (Quintana et al., 2011; Villalobos et al., 2018). This form of ROS-dependent regulation of ORAI1 channels remains controversial (Samanta et al., 2018; Samanta et al., 2019; Parekh, 2019).

In addition to aspirin and NSAIDs, other drugs have been reported to provide chemoprotection against several forms of cancer, particularly colorectal cancer. The most critical compound is DFMO, also called eflornithine. DFMO is considered a suicide inhibitor of ornithine decarboxylase 1 (ODC1), the critical enzyme in the biosynthesis pathway of the main mammalian polyamines: putrescine, spermine and spermidine. This enzyme is usually overexpressed in cancer cells, thus explaining the increased levels of polyamines that may contribute to cancer cell hallmarks, including enhanced cell proliferation, migration and invasion (Luk and Baylin, 1984; Pegg, 1988; Giardiello et al., 1997; Weiss et al., 2002). Several authors have reported ODC1 polymorphisms and robust correlation between specific polyamines and tissue growth (Martínez et al., 2003; Pegg, 2016; Wang et al., 2017; Sánchez-Jiménez et al., 2019). Finally, different growth stimuli including tumor promoters are able to enhance ODC activity and tumor formation (Bachmann and Geerts, 2018; Gerner et al., 2018). These effects are inhibited by DFMO, including inhibition of colon carcinogenesis in cancer models, such as the ApcMin/+ mice with high ODC levels and polyamines in the gastrointestinal tract. Interestingly, combinations of DFMO and NSAIDs have synergistic effects and inhibit dramatically carcinogenesis in both the small and large intestines of these mice. Recent clinical trials indicate that DFMO prevents CRC, especially if combined with sulindac, a carboxylic NSAID (Meyskens et al., 2008; Thompson et al., 2010; Burke et al., 2016).

Recent data reported by Gutierrez et al., suggest that DFMO may inhibit carcinogenesis by partially reversing the remodeling of store-operated Ca^2+^ channels in colorectal cancer cells (Gutiérrez et al., 2019). As stated above, colorectal cancers undergo a remodeling of intracellular Ca^2+^ homeostasis consisting in enhanced store-operated Ca^2+^ entry and decreased Ca^2+^ store content that contributed to cancer cell hallmarks, including enhanced cell proliferation, invasion and survival (Sobradillo et al., 2014; Villalobos et al., 2016; Villalobos et al., 2017). Villalobos and cols. have proposed that, during carcinogenesis, colorectal cancer cells may switch from a small, transient SOCE based on ORAI1-dependent CRAC channels (normal cells) to large and sustained currents depending on both ORAI1 and TRPC1 channels (tumor cells) (Villalobos et al., 2016; Villalobos et al., 2017; Villalobos et al., 2019). Interestingly, DFMO treatment inhibits cancer cell hallmarks in these cells including cell proliferation and cell death resistance. These effects are associated to SOCE inhibition and enhancement of Ca^2+^ store content. Therefore, DFMO may inhibit cancer cell hallmarks by reversing Ca^2+^ remodeling in colorectal cancer cells (Gutiérrez et al., 2019). Further research revealed that DFMO treatment inhibits TRPC1 expression and eliminates the TRPC1 component of the store-operated currents in colon cancer cells. These effects are reversed in the presence of polyamine putrescine (Gutiérrez et al., 2019). Collectively, these data suggest that polyamines promote Ca^2+^ channel remodeling by inducing expression of TRPC1 channels and this change promotes cancer cell hallmarks. In fact, this process may simply represent the exacerbation of the physiological process known as epithelial restitution that is mediated by transient polyamine synthesis induce by epithelial damage (Rao et al., 2012). While in physiological conditions this is a transient process limited by the repair of the wound epithelium, in cancer cells this process may became chronic due to ODC overexpression associated to activation of c-myc oncogene secondary to mutations in APC.

As stated above, combination of DFMO plus sulindac prevents efficiently colorectal cancer in high risk patients. An ongoing, large scale, three phase clinical trial is presently testing this treatment for patients at high risk of CRC (S0820, Adenoma and Second Primary Prevention Trial—Full Text View—ClinicalTrials.gov). Interestingly, Gutierrez et al. have reported that the combination of DFMO plus sulindac removes not only the TRPC1 component of the store-operated currents but also the ORAI1-dependent, thus fully abolishing the current. The mechanisms are different. The TRPC1-dependent current is removed at the expression level. However, the ORAI1-dependent current is simply inactivated because the mitochondria-dependent potentiation of the current is prevented. In summary, these data suggest a critical role for Ca^2+^ channel remodeling in cancer and provide a molecular mechanism of cancer chemoprevention targeting the remodeling of ion channels.

## Concluding Remarks

It is clearly evident in the literature, that Ca^2+^-permeable channels, transporters and pumps play important roles in a wide range of cancer-related process. The remodeling of these Ca^2+^-permeable channels contributes to the Ca^2+^-homeostasis dysregulation and both regulate several of the well-known cancer hallmarks. The influence of Ca^2+^ and other ion channels in carcinogenesis is so evident, that cancer was recently described as a channelopathy. This is a relative new field, however important advances in the identification of mechanism relating ion channels and cancer has been done. For this reason, ion channels represent a novel and possibly important clinical targets for some cancer types, with only a few channel blockers tested in clinical trials.

The use of ion channels modulators as chemotherapeutic agents has their pros and cons. Most of the Ca^2+^-permeable channels are ubiquitously expressed in different tissues, playing an important role in normal cell function. This is why, the pharmacological modulation of a specific cancer-related channel in tumor cells may produce significant toxicity in normal cells. Further research is required to take full advantage of channel modulators for cancer prevention, diagnosis and treatment.

## Author Contributions

Both authors contributed equally to the writing of the manuscript.

## Funding

This work has been supported by competitive grants RTI2018-099298-B-100 from Ministerio de Ciencia, Innovación y Universidades, Spain, BFU2015-70131 from Ministerio de Economía y Competitividad, Spain and VA294P18 from Junta de Castilla y León, Spain.

## Conflict of Interest

The authors declare that the research was conducted in the absence of any commercial or financial relationships that could be construed as a potential conflict of interest.
